# Supplementary material for: Acceptance and compliance with micronutrient powder (MNP) among children aged 6–23 months in northern Nigeria
Source: PLOS Glob Public Health. 2022 Oct 17;2(10):e0000961. doi: 10.1371/journal.pgph.0000961 (PMC10022258; doi:10.1371/journal.pgph.0000961)
Supplement: S1 Dataset — (ZIP) [file pgph.0000961.s008.zip › S8_Dataset/Spot Checks.zip!/Spot Checks/09072015_A_GA_ID_2_SCb.pdf]

# SPOT CHECK RECORDING FORM

**State: __Adamawa___ LGA: Ganye______ Community: Dabora_______**

**Name of caregiver (female): Fatima Hamman**

**Name of caregiver (Male):**

**Sex of index child: ___Female__ Age of index child (in months): 19 months_______**

## Spot Check Information

1. **Date of MNP distribution to household** (circle option below based on LGA name above)**:**
   - **Kebbi:** BirninKebbi&Jega LGA: **June 16**
   - **Kebbi:** Danko-wasagu: **June 17**
   - **Adamawa:**Song, Ganye: **June 18**
   - **Adamawa:**Guyuk **June 19**
2. **Date of Spot Check Observation: 09-07-2015_**
3. **Number of sachets remaining**(Ask the caregiver to show you and count the specific number of sachets remaining)**:**

   36

4. **Number of sachets that should remainusing one MNP sachet per day** (Analyst only)**:**

   40

5. **Number of sachets remaining / number of sachets should be remaining**(Analyst only)**:**

**___36______ / ___40______ = ____90_____ %**

## Probes

**Question 1.** You have **XX** MNP sachets remaining. Explain how easy or difficult it was to use the MNP product on a daily basis for your child 6 – 23 months? (Circle one answer)

**Very Difficult** **Difficult** **Easy** **Very Easy**

**Question 2.**Describe any specific challenges that you faced that made the MNP difficult to use on a daily basis?

At first when the child see MNP she don't like to take the pap but as time goes on we mix it in the room before coming out to feed the child.

**Question 3.** Describe specific aspects of the MNP product that you or your child enjoyed that made it easier to use on a daily basis?

The health change when we start giving the child MNP and it also make her to be eaten more food than before.

**Question 4.** What questions do you have about the MNP based on your experience using it?

**NIL**

**Question 5.** What specific recommendations do you have that we can do as a program to make the MNP easier to use on a daily basis?

The product make my child to be eaten food and sucks breast milk more than before.
